# Characterization of two *Arabidopsis thaliana *acyltransferases with preference for lysophosphatidylethanolamine

**DOI:** 10.1186/1471-2229-9-60

**Published:** 2009-05-16

**Authors:** Kjell Stålberg, Ulf Ståhl, Sten Stymne, John Ohlrogge

**Affiliations:** 1 Department of Plant Biology and Forest Genetics, Swedish University of Agricultural Sciences, Box 7080, SE-750 07 Uppsala, Sweden; 2 Department of Plant Breeding and Biotechnology, Swedish University of Agricultural Sciences, Box101, SE-230 53 Alnarp, Sweden; 3 Department of Plant Biology, Michigan State University, East Lansing, MI 48824, USA

## Abstract

**Background:**

Two previously uncharacterized Arabidopsis genes that encode proteins with acyltransferase PlsC regions were selected for study based on their sequence similarity to a recently identified lung lysophosphatidylcholine acyltransferase (LPCAT). To identify their substrate specificity and biochemical properties, the two Arabidopsis acyltransferases, designated AtLPEAT1, (At1g80950), and AtLPEAT2 (At2g45670) were expressed in yeast knockout lines *ale1 *and *slc1 *that are deficient in microsomal lysophosphatidyl acyltransferase activities.

**Results:**

Expression of AtLPEAT1 in the yeast knockout *ale1 *background exhibited strong acylation activity of lysophosphatidylethanolamine (LPE) and lysophosphatidate (LPA) with lower activity on lysophosphatidylcholine (LPC) and lysophosphatidylserine (LPS). AtLPEAT2 had specificities in the order of LPE > LPC > LPS and had no or very low activity with LPA. Both acyltransferases preferred 18:1-LPE over 16:0-LPE as acceptor and preferred palmitoyl-CoA as acyl donor in combination with 18:1-LPE. Both acyltransferases showed no or minor responses to Ca^2+^, despite the presence of a calcium binding EF-hand region in AtLPEAT2. AtLPEAT1 was more active at basic pH while AtLPEAT2 was equally active between pH 6.0 – 9.0.

**Conclusion:**

This study represents the first description of plant acyltransferases with a preference for LPE. In conclusion it is suggested that the two AtLPEATs, with their different biochemical and expression properties, have different roles in membrane metabolism/homoeostasis.

## Background

Acyltransferases comprise several families of enzymes with diverse origins and functions. The first acyltransferase recognized to be involved in phosphatydylethanolamine synthesis was identified based on the PlsC mutant in *Escherichia coli*, (named after its defect in phospholipid synthesis). Over 5000 acyltransferses are annotated in the UniProtKB database with a PlsC signature [1]. In the Arabidopsis genome, 24 proteins are encoded that contain this signature but the substrate specificity has been determined for less than 10 members. Most of the PlsC domain acyltransferases have two conserved domains NH(X)_4_D, (IPB002123A) [2] and FPEGT, (IPB002123B) [2] of which the former is also part of the active site. This broad family of enzymes accept at least two different acyl donors, acyl-CoA and acyl-ACP and several acyl acceptors such as glycerol-3-phosphate (G3P), and lysophospholipids and include members of 1-acyl-sn-glycerol-3-phosphate acyltransferase, tafazzin and glycerol-3-phosphate O-acyltransferase enzymes (IPR002123). Lysophosphatidic acid acyltransferases (LPAATs) of the PlsC type have been cloned and characterised from many organisms. Much more scarcely represented are lysophosphatidylethanolamine specific acyltransferses (LPEATs). However, a bifunctional acyl-ACP synthase having an acyltransferase domain of the PlsC type was shown to be involved in uptake and acylation of 1-acyl-glycerophosphoethanolamine, (1-acyl-GPE), in *E. coli*, [3]. Moreover, in *Synechocystis *a broad acyl-ACP specificity acyltransferase with 1-acyl-glycerophosphoethanolamine activity, and with a strong preference for 16:0-ACP/lysophosphatidylglycerol, (LPG) has been well characterized, [4]. Very recently a human LPEAT2 was cloned and shown to be expressed in brain [5].

In yeast, *Saccharomyces cerevisiae*, a novel type of acyltransferases YOR175c (*ALE1*) belonging to the family of membrane bound O-acyltransferases (MBOAT) has recently been shown to represent a major acyl-CoA dependent lysophospholipid acyltransferase [6-12]. Knockout of *ALE1 *(Δ*yor175c:KanMX4) *results in the loss of *in vitro *acylation of LPC, LPE, LPI, LPG, LPS and LPA, but sufficient activity of LPAAT is still provided for *de novo *synthesis of phospholipids by *SLC1*, (PlsC type). While knockout of either *ALE1 *or *SLC1 *results in no obvious growth defect, double knockout of *ale1/slc1 *results in a severe growth defect [13] or lethality [6,8,12], which suggests that together the two enzymes are indispensable for the synthesis of PA. A novel plant LPAAT was identified by functional complementation of the *Escherichia coli *mutant PlsC by Bourgis et al., [14] and in Arabidopsis a plastidic LPAAT was shown to be essential for embryo development [15,16]. By analysis of the Arabidopsis genome and alignments of putative acyltransferases in a phylogenetic tree, fifteen Arabidopsis genes encoding proteins that had both NH(X)_4_D and EGT motifs were identified [15]. Among them, At1g80950 was found, but to our knowledge was not further characterized. A second gene at locus At2g45670, less similar, was not identified, although it belongs to the same family of genes. In this study we have expressed cDNAs from these two genes in yeast *ale1 *and *slc1 *knockouts and initially characterized their biochemical functions *in vitro*.

## Results

**Figure 1:**
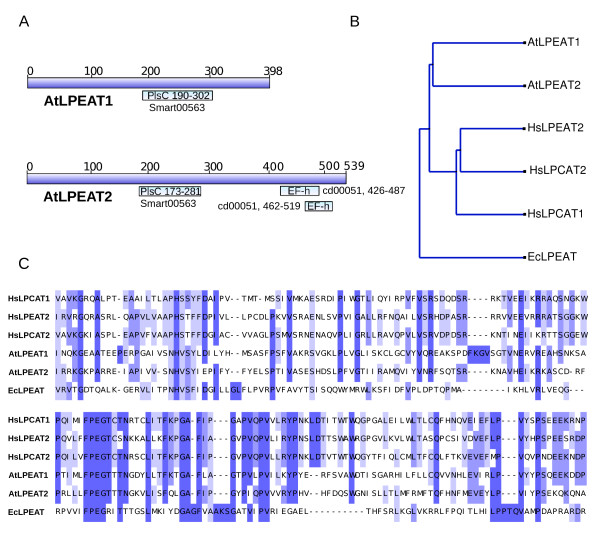
**Schematic drawings showing binding sites, relationship and alignment of different acyltransferases**. A. Schematic drawing showing the two AtLPEATs indicating PlsC and putative Ca^2+ ^binding sites, (cd00051). B. Tree representing relationship between AtLPEAT1, AtLPEAT2, *Escherichia coli Ec*LPEAT aas gene [3], HsLPEAT2, [5], HsLPCAT1 and HsLPCAT2 [24], was produced with jalview using percentage identity over shown region. C. Part of a Kalign alignment (default settings, EBI), of the three proteins showing conserved PlsC domain motifs NHX(4)D, FPEGT and indicated conserved amino acids.

Two uncharacterised proteins from Arabidopsis, (locus At1g80950 and At2g45670) were identified based upon their predicted amino acid sequence similarity to mouse lung lysophosphatidylcholine acyltransferase (translated sequence of accession number AB244717). Both proteins possess an acyltransferase (PlsC) domain, and one to several transmembrane spanning regions. Only the candidate protein At2g45670 was predicted to have an EF-hand/calcineurin calcium binding domain. A schematic drawing of the two proteins is shown in Figure 1A. None of the candidates scored high for a particular subcellular localisation, but both were predicted to be in the secretory pathway using Aramemnon topology consensus search, [17]. AtLPEAT1 was also ER predicted in a study mapping the Arabidopsis proteome [18]. Analysis by AtGenExpress indicated that mRNA from both genes were expressed at low levels in most tissues with slightly higher expression in flower. Expression of At1g80950 was higher in the seed, while the At2g45670 signal was lower at later stages of seed development, TAIR, (AtGenExpress visualisation tool), [19]. As the codon usage of both cDNAs did not largely deviate from optimized yeast codon tables, the cDNAs were expressed in yeast to initially characterize their substrate specificity and biochemical characteristics. Because of strong endogenous lysophospholipid acyltransferase activities in WT yeast, this study took advantage of the mutants of the lysophospholipid acyltransferases *ALE1 *(Δ*yor175c:KanMX4) *and *SLC1*, (Δ*ydl052c:KanMX4*) to express these novel Arabidopsis acyltransferases.

Expression of the two putative lysophospholipid acyltransferases AtLPEAT1, (At1g80950) and AtLPEAT2, (At2g45670) in yeast *ale1 *showed that both proteins possess acyltransferase activity with long-chain acyl-CoA and lysophospholipid substrates. Time and protein dependence of reactions were determined for AtLPEAT2 with 18:1-CoA and 18:1-LPE. The correlation coefficients were 0.94, (nmol/mg/0–2 min) and 0.98, (nmol/0.5 – 2 mg/min) between the variables, indicating good linearity of the assay [see Additional file 1 and 2].

**Figure 2:**
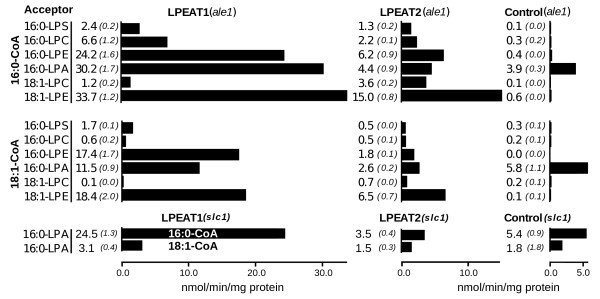
**Acyltransferase activity from transformants expressing either LPEAT1, LPEAT2 or control empty vector**. The data represent triplicate samples with products separated on TLC plates and quantified by Instant Imager autoradiography. The mean values are presented to the left of each horizontal bar with standard error in parenthesis and in italics. The concentration of 16:0-CoA and 18:1-CoA were 22.7 μM in all assays except for the assay with 16:0-LPA as an acceptor in the yeast *slc1 *strain, which were 2.25 nmol of [^14^C]18:1-CoA (75000 dpm). The amount of microsomal protein added to the assays of the *ale1 *transformants were: for LPS, 1.88 μg, LPC and LPE, 1.33 μg, and LPA, 1.16 μg, and in the assay of LPA as an acceptor in the *slc1 *transformants, LPEAT1, LPEAT2 and empty vector control, 2, 2.08, 1.5 μg respectively.

Based on the sequence similarity of the Arabidopsis proteins to the mouse lung LPCAT, we first tested the acylation of 16:0-LPC and found that this substrate was indeed an acceptor for the two enzymes, whereas 18:1-LPC was a relatively poor acyl acceptor when the acyl donor was 18:1-CoA, (Figure 2). The highest activities in these assays were seen with the combination of 16:0-LPC and 16:0-CoA for AtLPEAT1 whereas AtLPEAT2 preferred 18:1-LPC with 16:0-CoA. The empty vector control reflects the phenotype of *ale1*, which was well below the acylation rate of LPC of lines expressing AtLPEAT1 or AtLPEAT2 for most substrates (Figure 2). Acylation activities in the LPEAT transformants without addition of any lysophospholipid acceptor indicated there were endogenous lysophospholipids in microsomal extracts [see Additional file 3]. This background activity gave hints on which substrates were preferred by the two enzymes, but the molecular species of these acyl acceptors might not represent acyl acceptors in Arabidopsis tissues.

Assays with 16:0-LPE and 18:1-LPE with different combinations of 16:0-CoA and 18:1-CoA showed that both Arabidopsis acyltransferases were more active with these acyl acceptors than with LPC (Figure 2). AtLPEAT1 was more active with both LPE substrates than AtLPEAT2. Both enzymes had highest activity with 16:0-CoA and 18:1-LPE. Di-18:1-PE and di-16:0-PE were equally well synthesized as by AtLPEAT2 (Figure 2).

**Figure 3:**
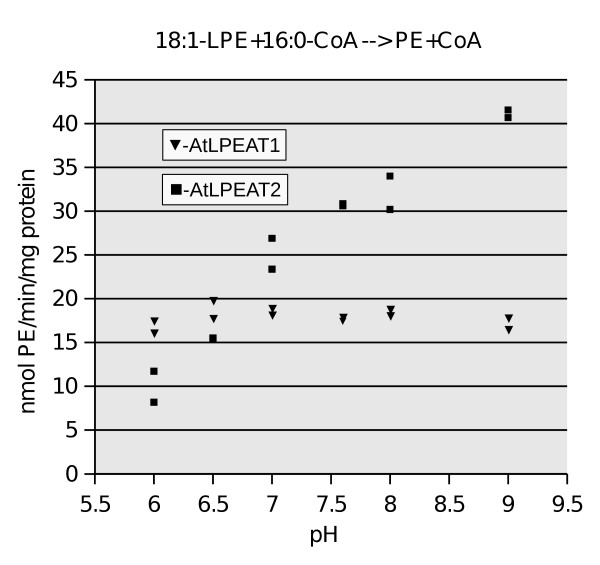
**pH dependency of LPEAT1 and LPEAT2 activities**. Each assay contained 1.5 μg microsomal protein of yeast (*ale1*) from transformants expressing either LPEAT1, LPEAT2 or control empty vector.

A faint spot of PA was also an identified product when no acyl acceptor was added [see Additional file 3]. As *ale1 *has considerable endogenous LPAAT activity we also expressed the two Arabidopsis acyltransferase in *slc1 *to allow more reliable LPAAT assays. As shown in Figure 2, AtLPEAT1 had substantial LPAAT activities whereas AtLPEAT2 only exhibited activity just above background. The best acyl donor was 16:0-CoA in both yeast knockout backgrounds and there was a slight difference in acyl preference in the control reactions in two backgrounds (*ale1 *and *slc1)*, presumably reflecting the different specificities of yeast acyltransferases *SLC1 *in *ale1 *and ALE1 in *slc1*, (Figure 3). 16:0-LPS was a poor substrate for acylation by either of the two AtLPEATs, although substantially above background, and both enzymes showed similar preference for 16:0-CoA as an acyl donor over 18:1-CoA (Figure 2).

The possibility that the C-terminal EF-hand calcium binding domain provided regulation by calcium was tested by adding CaCl_2 _to the assay mixtures. However, neither of the two AtLPEATs activities was dramatically affected by this addition nor by EDTA [see Additional file 4]. Assaying pH dependency of the two Arabidopsis constructs expressed in yeast *ale1 *was done in 25 mM Tris buffer. In this analysis, AtLPEAT1 exhibited an almost linear increase in response at pH 6.0 – 9.0, while AtLPEAT2 had constant activities over this same pH interval (Figure 3).

## Discussion

Our initial attempt to characterise AtLPEAT1 and AtLPEAT2 by expression in wild-type yeast was obscured due to high background lysophosphatidylcholine acyltransferase activities of *ALE1 *LPCAT. However, recent cloning and characterisation of *ALE1 *in yeast made it possible to characterize our candidate proteins in a low background strain containing a kanMX4 knockout of this gene. Since *ale1 *has almost no lysophospholipid acyltransferase activities except for LPAAT, [6-8,10-12], expression in this mutant should reflect the substrate specificities of the candidate acyltransferases. However as one of the Arabidopsis enzymes turned out to have strong dual activity with high LPAAT activity, we also decided to express the Arabidopsis acyltransferases in the yeast, *SLC1 *knockout. The *SLC1 *gene was cloned as a suppressor gene which enabled yeast to grow without sphingolipid synthesis and was shown to code for an LPAAT protein [20]. Utilizing these yeast mutants for gal induced expression of AtLPEAT1 and AtLPEAT2 we found a remarkable difference in preference for LPA between the two enzymes. AtLPEAT1 preferred 16:0-CoA eight fold over 18:1-CoA using 16:0-LPA as an acyl acceptor. The increase of LPAAT activity was more pronounced in *slc1 *(8 fold), than in *ale1 *(2.6 fold). This selectivity of 16:0-CoA is somewhat similar to the Arabidopsis LPAAT1 (LPAT1) preference, although the acyl acceptor in their assay was 18:1-LPA, [15]. Four other Arabidopsis LPAATs (LPAT2, LPAT3, LPAT4 and LPAT5) were later isolated and characterized for their acyl donor specificities [21]. All four LPAATs possessed higher *in vitro *activity with 18:1-CoA than 16:0-CoA when expressed in wild-type yeast. AtLPEAT1 exhibited strong selectivity depending on the combination of acyl acceptor and acyl donor in the acylation of LPE and LPA. The best acceptor was 18:1-LPE in combination with 16:0-CoA. AtLPEAT2 lacked acylation capability of LPA except for a low rate of formation of di-16:0-PA. Like AtLPEAT1, AtLPEAT2 showed the highest specific activity with 18:1-LPE in combination with 16:0-CoA.

The sequence homology of AtLPEATs to mammalian LPCATs is intriguing (Figure 1B and 1C). An alignment over the region containing the PlsC domain of these evolutionarily distantly related proteins distinguishes these acyltransferases from other PlsC domain containing proteins distal of the FPEGT site. Using the FPEGT motif and an adjacent 30 aa of AtLPEAT1 in a BLAST search of the non-redundant protein database retrieved exclusively this family. As can be seen in Figure 1C AtLPEAT2 has a Glutamate (E) instead of the otherwise completely conserved Aspartate (D) in the catalytic site. The mouse lung LPCAT1, highly homologous to human, (Aytl2) and mouse/rat LPCAT, has been shown to have preference for saturated fatty acids of both acceptors and donors [22,23], while Aytl3 a human LPEAT2 showed no such preference for acyl-CoAs [5]. LPE was also a substrate for this enzyme, but in contrast to the two AtLPEATs it preferred LPC over LPE. The pH optimum for the mouse lung LPCAT was between 7.4 and 10, and the reaction did not require Ca^2+^. Neither of the two AtLPEATs required Ca^2+^. In contrast, the human HsLPCAT (Aytl2) exhibiting EF1 and EF2-hand domains as AtLPEAT2, was shown to be inhibited by Ca^2+^, [24] while HsLPEAT2 was not effected by Ca^2+ ^[5]. AtLPEAT1 had a completely different response to pH 6.0 – 9.0 while AtLPEAT2 resembled the human LPCATs by being non sensitive within that pH range.

In eukaryotes, synthesis of PE occurs by three different pathways. Decarboxylation of PS, phosphoethanolamine transfer from CDP-ethanolamine to diacylglycerol and base exchange of the headgroup, (serine with ethanolamine). In Arabidopsis, CTP:phosphorylethanolamine cytidylyltransferase, (PECT) mutants exhibit a decrease of phosphatidylethanolamine and increase in phosphatidylcholine and embryo abortion, [25]. Both AtLPEATs were predicted to be in the secretory pathway and PE is known to be a rather specific target for plant secretory phospholipases A_2 _(PL A_2_) [26]. This type of phospholipase requires Ca^2+ ^for activity and has no acyl chain preference [26]. Auxin has been shown to induce and increase the levels of free fatty acids, LPC and LPE in microsomes [27] and LPE is also known to retard senescence, which might be related to LPE's inhibitory effect of phospholipase D [28]. Overexpression of AtsPL A_2 _in Arabidopsis results in prolonged leaf petiols and inflorescence stems and general cell elongation [29]. Whether the AtLPEATs described in this study have a function in relation to PL A_2_on induced responses or in organogenesis remains to be seen. Related to developmental cues is the generation of fatty acids for membrane synthesis. *In vitro *acylation of PC and PE in Arabidopsis showed that the ratio of labelling of PE to PC was highly different in root and leaf and specific PE remodelling by acylation has been demonstrated to be the cause of the remodelling [30], AtLPEAT1 and AtLPAT2 are obvious candidates involved in such remodelling.

The specific activity (10–30 nmol product/min/mg protein) of the LPEATs in the yeast microsomes were similar or higher than other lysophosphatidyl enzymes assayed under similar conditions. The acyl acceptors used in our assays were *sn-1-acyl *lysophospholipids and thus acylation occurred at the *sn-2 *position. It should be noted that palmitoyl-CoA was found to be the best acyl donor by the two AtLPEATs despite the fact that palmitate is primarily confined to the *sn-1 *position of plant PE. There are substantial problems to assay the *sn-1 *acylation activity of lysophospholipid acyltransferases because the sn-2 lysophospholipid substrates are unstable due to rapid acyl migration to the *sn-1 *position. Since the positional specificity of the LPEATs were not studied in this work, we can not infer any positional preference of the enzymes and cannot rule out that an additional *in-vivo *acyl acceptor for the AtLPEATs in plants might be *sn-2-*acyl lysophospholipids.

## Conclusion

In conclusion, backward transacylation reaction, [31] and/or hydrolysis of phospholipids in combination with forward lysophosphatidyl acyltransferases reaction could at least partly be maintained by the AtLPEATs described herein. The difference in expression pattern of the two AtLPEATs, together with their different biochemical properties, suggest that the two AtLPEAT homologues have partly different functions in membrane metabolism/homoeostasis. Future studies of Arabidopsis that are mutated or have overexpressed LPEAT genes are likely to give valuable information about their physiological functions.

## Methods

### Constructs and Strains

Two full-length cDNA clones (pda09299, pda10945) corresponding to the Arabidopsis genes At1g80950 and At2g45670 were obtained from RIKEN, [32]. Expression of the protein coding sequence of these clones in yeast using the pYES2 gal inducible system was enabled by PCR amplification, using *Pfu *polymerase under standard conditions. The following oligonuceotide primers were used for amplification: At1g80950 (1) 5'gtgggtaccataatggaatcagagctcaa-3', At1g80950 (2) 5' ccaggcatgctcattcttctttctgatggaa-3', At2g45670 (1) 5' cagggtaccagaatggcggatcctgatct-3', At2g45670 (2) 5' tctcgcatgcttatgttggggccaagtcag-3'. The oligonucleotides contained 5' *Kpn*1 and 3' *Sph*1 3' restriction sites for cloning into the corresponding sites of pYES2. The generated clones in pYES2 were sequenced to confirm identity with the cDNAs and were named AtLPEAT1 (At1g80950) and AtLPEAT2 (At2g45670). Two different haploid knockout mutants of *ALE1*, *ale1*:(BY4741; Mat a; his3Δ1; leu2Δ0; lys2Δ0; ura3Δ0; YOR175c::kanMX4) and *SLC1*, *slc1*:(BY4742; Mat α; his3Δ1; leu2Δ0; lys2Δ0; ura3Δ0; YDL052c::kanMX4) a corresponding wt: (BY4742; Mat α; his3Δ1; leu2Δ0; lys2Δ0; ura3Δ0). The yeast BY-series were from euroscarf [33] and were transformed to achieve expression of the two Arabidopsis acyltransferase proteins. An empty vector pYES2 was used as a control.

### Microsomal preparations

Transgenic yeast strains containing vector constructs encoding the acyltransferases and empty vector control were pre-cultured over night in 3 ml of synthetic media containing 2% Gal without uracil. Thirty ml of synthetic media containing 2% Gal -U was inoculated from the pre-culture (all diluted to the same OD) and grown at 28°C overnight to an OD of approximately 4.0. Twenty ml of fresh media were added to cultures and incubated for an additional 4 h. The cells were spun down in 50 ml falcon tubes and the supernatant discarded. The pellets were resuspended in 3 ml extraction buffer, (20 mM Tris-HCl pH 7.6, 1 mM EDTA) and transferred to 2 ml microfuge snap cap tubes containing approximately 0.5 ml of 0.5 mm zirconia silicabeads and the cells were disrupted in a Retch bead beater 30/s, 1 × 3 minutes in pre-chilled supplies. The homogenates were centrifuged at 1000 × g for 10 min and supernatants transferred to new tubes. The tubes were washed by adding 0.85 ml extraction buffer, vortexed, centrifuged and pooled with supernatants. The supernatants were diluted with extraction buffer to 8 ml and spun at 100 000 × g, 1 hour 4°C. The pellet was carefully resuspended in 0.5 ml extraction buffer, and aliquots of 50 μl extracts, "microsomal extracts", were frozen and stored at -70°C.

### Lipid analysis and assay conditions

Protein concentrations were determined by Bradford assay using fatty acid free BSA as a standard. The assay for acyltransferase reaction (final volume of 33 μl) contained, if not otherwise stated in the Figure legends; 25 mM tris-HCl pH 7.6, 0.4 M KCl, 0.75 nmol [1-^14^C]acyl-CoA (25000 dpm), obtained from American Radiolabeled Chemicals, St Louis, 0.25 nmol of synthetic lysophospholipids (Avanti polar lipids, inc) and microsomal preparations as amounts indicated in the Figures. 0.4 M KCl was added to avoid precipitation of acyl-CoA as cations precipitate most of palmitoyl-CoA at mM concentration. Lysophospholipids dissolved in water/ethanol 1:1, were heated briefly before adding to the assay mix. The reactions were started by adding "microsomal extract" preparations under vortexing to pre-warmed microfuge tubes containing the assay mix. The reaction was incubated at 30°C for 2 minutes and stopped by adding 133 μl 50:50:1, methanol/chloroform/acetic acid and vortexing. After addition of 13.3 μl H_2_O the mixture was, vortexed, centrifuged 13000 × g and the lower phase was transferred to new tube. The water phase was extracted once again by adding 66 μl chloroform. The pooled organic phases were evaporated under a stream of N_2_, dissolved in 15 μl chloroform and spotted on silica gel 60, 20 × 20 cm TLC plates (Merck). The TLC plates were developed to 3/4 in 85:15:10:3.5 methanol/chloroform/acetic acid/water. The phospholipids were identified by comparison to known standards and quantification was done by autoradiography on a Packard InstantImager, with Version 2.05 software, using triplicates of 1/10^th ^of the assay mix, (without microsomal preparations), spotted after development at top of each plate. Background subtraction was done for each lane at positions where no visible spots could be seen. Synthetic lysophospholipid acyl acceptors were; 1-palmitoyl-2-hydroxy-sn-glycero-3-phosphocholine, 1-oleoyl-2-hydroxy-sn-glycero-3-phosphocholine, 1-palmitoyl-2-hydroxy-sn-glycero-3-phosphoethanolamine, 1-oleoyl-2-hydroxy-sn-glycero-3-phosphoethanolamine, 1-palmitoyl-2-hydroxy-sn-glycero-3-phosphatidate, 1-palmitoyl-2-hydroxy-sn-glycero-3-phosphoserine. Acyl donors were; palmitoyl-Coenzyme A, (16:0-CoA), oleoyl-Coenzyme A, (18:1-CoA) and corresponding radiolabeled acyl-CoAs; [^14^C]16:0-CoA 60 mCi/mmol 0.02 mCi/ml [^14^C ]18:1-CoA 55 mCi/mmol 0.1 mCi/ml.

## Authors' contributions

KS carried out the biochemical and molecular genetic analyses. All authors gave ideas, revised, read and approved the final manuscript. JO coordinated the study.

## Supplementary Material

Additional File 1 **Time-course of LPEAT2 activity using 18:1-CoA and 18:1-LPE, with 1.22 μg microsomal protein of a yeast (*ale1 *strain) transformant expressing LPEAT2**. Time point at zero represent a single reaction directly stopped by adding 133 μl 1:1:50, methanol/chloroform/acetic acid before adding the microsomal extract. The correlation coefficient for the variables was 0.94. non-standard format. Click here for file

Additional File 2 **Acylation of 18:1-LPE by LPEAT2 as a function of protein concentration**. Assays were performed at indicated concentrations of microsomal protein of yeast (*ale1 *strain) transformant expressing LPEAT2. The correlation coefficient for the variables was 0.98. Click here for file

Additional File 3 **Formation of phospholipids without addition of acyl acceptors**. The acylation of endogenouslysophospholipids was assayed using microsomal preparations from LPEAT1 (*ale1) *and LPEAT2 (*ale1) *using either 16:0-CoA or 18:1-CoA as acyl donors. Data are the means of triplicates of indicated phosphorimager detected phospholipids calculated as nmol/min/mg protein. Numbers in parenthesis are sample standard errors. Click here for file

Additional File 4 **The effect of cations and EDTA on LPEAT1 and LPEAT2 activities**. The assays were performed by pre-incubating; 2 mM ZnCl_2_; 2 mM CaCl_2 _or 5 mM EDTA with 1.5 μg microsomal protein of yeast (*ale1 *strain) from transformants expressing either LPEAT1 or LPEAT2 for 10 minutes at ambient temperature prior to the addition of the enzyme substrates. Error bars indicate standard error of the sample means of triplicate measurements. Click here for file

## References

[B1] UniProtKB, search PlsC. http://www.uniprot.org/.

[B2] InterPro, search IPR002123. http://www.ebi.ac.uk/interpro/.

[B3] Jackowski S, Jackson P, Rock C (1994). Sequence and function of the aas gene in *Escherichia coli*. J Biol Chem.

[B4] Weier D, Müller C, Gaspers C, Frentzen M (2005). Characterisation of acyltransferases from Synechocystis sp PCC6803. Biochem Biophys Res Commun.

[B5] Cao J, Shan D, Revett T, Li D, Wu L, Liu W, Tobin JF, Gimeno RE (2008). Molecular identification of a novel mammalian brain isoform of acyl-CoA:lysophospholipid acyltransferase with prominent ethanolamine lysophospholipid acylating activity, LPEAT2. J Biol Chem.

[B6] Benghezal M, Roubaty C, Veepuri V, Knudsen J, Conzelmann A (2007). SLC1 and SLC4 encode partially redundant acyl-coenzyme A 1-acylglycerol-3-phosphate O-acyltransferases of budding yeast. J Biol Chem.

[B7] Chen Q, Kazachkov M, Zheng Z, Zou J (2007). The yeast acylglycerol acyltransferase LCA1 is a key component of Lands cycle for phosphatidylcholine turnover. FEBS Lett.

[B8] Jain S, Stanford N, Bhagwat N, Seiler B, Costanzo M, Boone C, Oelkers P (2007). Identification of a novel lysophospholipid acyltransferase in *Saccharomyces cerevisiae*. J Biol Chem.

[B9] Riekhof WR, Wu J, Gijón MA, Zarini S, Murphy RC, Voelker DR (2007). Lysophosphatidylcholine metabolism in Saccharomyces cerevisiae: the role of P-type ATPases in transport and a broad specificity acyltransferase in acylation. J Biol Chem.

[B10] Riekhof WR, Wu J, Jones JL, Voelker DR (2007). Identification and characterization of the major lysophosphatidylethanolamine acyltransferase in *Saccharomyces cerevisiae*. J Biol Chem.

[B11] Ståhl U, Stålberg K, Stymne S, Ronne H (2008). A family of eukaryotic lysophospholipid acyltransferases with broad specificity. FEBS Lett.

[B12] Tamaki H, Shimada A, Ito Y, Ohya M, Takase J, Miyashita M, Miyagawa H, Nozaki H, Nakayama R, Kumagai H (2007). LPT1 encodes a membrane-bound O-acyltransferase involved in the acylation of lysophospholipids in the yeast *Saccharomyces cerevisiae*. J Biol Chem.

[B13] Schuldiner M, Collins SR, Thompson NJ, Denic V, Bhamidipati A, Punna T, Ihmels J, Andrews B, Boone C, Greenblatt JF, Weissman JS, Krogan NJ (2005). Exploration of the function and organization of the yeast early secretory pathway through an epistatic miniarray profile. Cell.

[B14] Bourgis F, Kader JC, Barret P, Renard M, Robinson D, Robinson C, Delseny M, Roscoe TJ (1999). A plastidial lysophosphatidic acid acyltransferase from oilseed rape. Plant Physiol.

[B15] Kim HU, Huang AHC (2004). Plastid lysophosphatidyl acyltransferase is essential for embryo development in Arabidopsis. Plant Physiol.

[B16] Yu B, Wakao S, Fan J, Benning C (2004). Loss of plastidic lysophosphatidic acid acyltransferase causes embryo-lethality in Arabidopsis. Plant Cell Physiol.

[B17] Aramemnon. http://aramemnon.botanik.uni-koeln.de/indexep.

[B18] Dunkley TPJ, Hester S, Shadforth IP, Runions J, Weimar T, Hanton SL, Griffin JL, Bessant C, Brandizzi F, Hawes C, Watson RB, Dupree P, Lilley KS (2006). Mapping the Arabidopsis organelle proteome. Proc Natl Acad Sci USA.

[B19] TAIR. http://www.arabidopsis.org/.

[B20] Nagiec MM, Wells GB, Lester RL, Dickson RC (1993). A suppressorgene that enables *Saccharomyces cerevisiae *to grow withoutmaking sphingolipids encodes a protein that resembles an *Escherichia coli *fatty acyltransferase. J Biol Chem.

[B21] Kim HU, Li Y, Huang AHC (2005). Ubiquitous and endoplasmic reticulum-located lysophosphatidyl acyltransferase, LPAT2, is essential for female but not male gametophyte development in Arabidopsis. Plant Cell.

[B22] Nakanishi H, Shindou H, Hishikawa D, Harayama T, Ogasawara R, Suwabe A, Taguchi R, Shimizu T (2006). Cloning and characterization of mouse lung-type acyl-CoA:lysophosphatidylcholine acyltransferase 1 (LPCAT1) Expression in alveolar type II cells and possible involvement in surfactant production. J Biol Chem.

[B23] Chen X, Hyatt BA, Mucenski ML, Mason RJ, Shannon JM (2006). Identification and characterization of a lysophosphatidylcholine acyltransferase in alveolar type II cells. Proc Natl Acad Sci USA.

[B24] Soupene E, Fyrst H, Kuypers FA (2008). Mammalian acyl-CoA:lysophosphatidylcholine acyltransferase enzymes. Proc Natl Acad Sci USA.

[B25] Mizoi J, Nakamura M, Nishida I (2006). Defects in CTP:phosphorylethanolamine cytidylyltransferase affect embryonic and postembryonic development in Arabidopsis. Plant Cell.

[B26] Bahn SC, Lee HY, Kim HJ, Ryu SB, Shin JS (2003). Characterization of Arabidopsis secretory phospholipase A _2_-gamma cDNA and its enzymatic properties. FEBS Lett.

[B27] Roland U, Holk Paul andré, Scherer GüntherFE (1998). Fattyacids and lysophospholipids as potential second messengers in auxin action Rapid activation of phospholipase A_2 _activity by auxin in suspension-cultured parsley and soybean cells. The Plant Journal.

[B28] Ryu SB, Karlsson BH, Ozgen M, Palta JP (1997). Inhibition of phospholipase D by lysophosphatidylethanolamine, a lipid-derived senescence retardant. Proc Natl Acad Sci USA.

[B29] Lee HY, Bahn SC, Kang Y, Lee KH, Kim HJ, Noh EK, Palta JP, Shin JS, Ryu SB (2003). Secretory low molecular weight phospholipase A2 plays important roles in cell elongation and shoot gravitropism in Arabidopsis. Plant Cell.

[B30] Hocquellet A, Joubès J, Perret A, Lessire R, Moreau P (2005). Evidence for a different metabolism of PC and PE in shoots and roots. Plant Physiol Biochem.

[B31] Stymne S, Stobart AK (1984). Evidence for the reversibility of the acyl-CoA:lysophosphatidylcholine acyltransferase in microsomal preparations from developing safflower (*Carthamus tinctorius *L.) cotyledons and rat liver. Biochem J.

[B32] RIKEN. http://www.brc.riken.jp/lab/epd/catalog/cdnaclone.html.

[B33] Euroscarf, Institute of Microbiology, University of Frankfurt, Germany. http://web.uni-frankfurt.de/fb15/mikro/euroscarf/.

