# Supplementary material for: Characterization of two Arabidopsis thaliana acyltransferases with preference for lysophosphatidylethanolamine
Source: BMC Plant Biol. 2009 May 16;9:60. doi: 10.1186/1471-2229-9-60 (PMC2690597; doi:10.1186/1471-2229-9-60)
Supplement: Additional File 1 — Time-course of LPEAT2 activity using 18:1-CoA and 18:1-LPE, with 1.22 μg microsomal protein of a yeast (ale1 strain) transformant expressing LPEAT2. Time point at zero represent a single reaction directly stopped by adding 133 μl 1:1:50, methanol/chloroform/acetic acid before adding the microsomal extract. The correlation coefficient for the variables was 0.94. non-standard format. [file 1471-2229-9-60-S1.pdf]

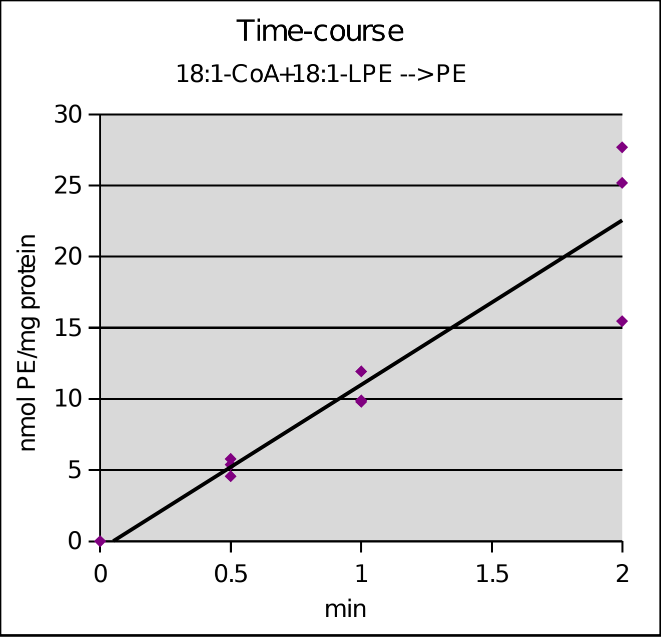

Time-course of LPEAT2 activity using 18:1-CoA and 18:1-LPE, with 1.22 µg microsomal protein of a yeast (ale1 strain) transformant expressing LPEAT2. Time point at zero represent a single reaction directly stopped by adding 133 µl 1:1:50, methanol/ chloroform/ acetic acid before adding the microsomal extract. The correlation coefficient for the variables was 0.94. non-standard format.
