# Supplementary material for: Characterization of two Arabidopsis thaliana acyltransferases with preference for lysophosphatidylethanolamine
Source: BMC Plant Biol. 2009 May 16;9:60. doi: 10.1186/1471-2229-9-60 (PMC2690597; doi:10.1186/1471-2229-9-60)
Supplement: Additional File 2 — Acylation of 18:1-LPE by LPEAT2 as a function of protein concentration. Assays were performed at indicated concentrations of microsomal protein of yeast (ale1 strain) transformant expressing LPEAT2. The correlation coefficient for the variables was 0.98. [file 1471-2229-9-60-S2.pdf]

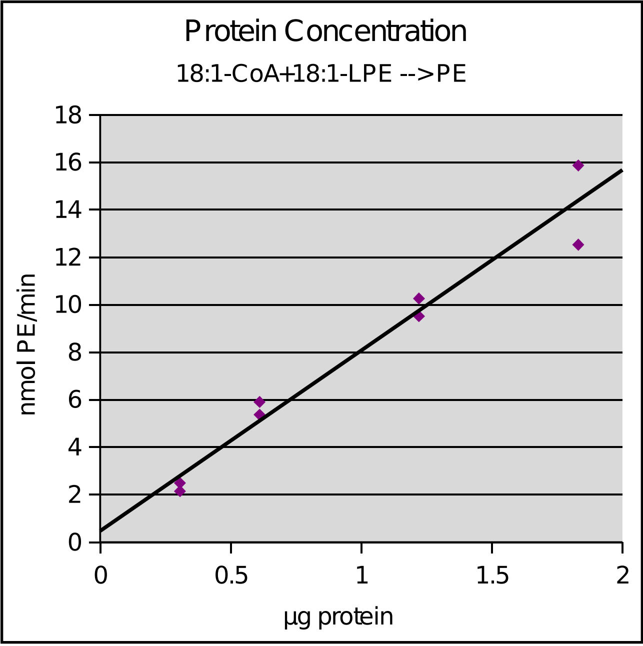

Acylation of 18:1-LPE by LPEAT2 as a function of protein concentration. Assays were performed at indicated concentrations of microsomal protein of yeast (*ale1* strain) transformant expressing LPEAT2. The correlation coefficient for the variables was 0.98.
