# Supplementary material for: Characterization of two Arabidopsis thaliana acyltransferases with preference for lysophosphatidylethanolamine
Source: BMC Plant Biol. 2009 May 16;9:60. doi: 10.1186/1471-2229-9-60 (PMC2690597; doi:10.1186/1471-2229-9-60)
Supplement: Additional File 3 — Formation of phospholipids without addition of acyl acceptors. The acylation of endogenouslysophospholipids was assayed using microsomal preparations from LPEAT1 (ale1) and LPEAT2 (ale1) using either 16:0-CoA or 18:1-CoA as acyl donors. Data are the means of triplicates of indicated phosphorimager detected phospholipids calculated as nmol/min/mg protein. Numbers in parenthesis are sample standard errors. [file 1471-2229-9-60-S3.pdf]

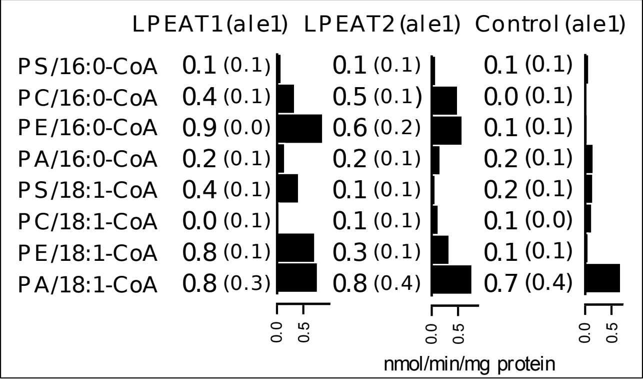

Formation of phospholipids without addition of acyl acceptors. The acylation of endogenous lysophospholipids was assayed using microsomal preparations from LPEAT1 (ale1) and LPEAT2 (ale1) using either 16:0-CoA or 18:1-CoA as acyl donors. Data are the means of triplicates of indicated phosphorimager detected phospholipids calculated as nmol/min/mg protein. Numbers in parenthesis are sample standard errors.
