# Supplementary material for: Characterization of two Arabidopsis thaliana acyltransferases with preference for lysophosphatidylethanolamine
Source: BMC Plant Biol. 2009 May 16;9:60. doi: 10.1186/1471-2229-9-60 (PMC2690597; doi:10.1186/1471-2229-9-60)
Supplement: Additional File 4 — The effect of cations and EDTA on LPEAT1 and LPEAT2 activities. The assays were performed by pre-incubating; 2 mM ZnCl2; 2 mM CaCl2 or 5 mM EDTA with 1.5 μg microsomal protein of yeast (ale1 strain) from transformants expressing either LPEAT1 or LPEAT2 for 10 minutes at ambient temperature prior to the addition of the enzyme substrates. Error bars indicate standard error of the sample means of triplicate measurements. [file 1471-2229-9-60-S4.pdf]

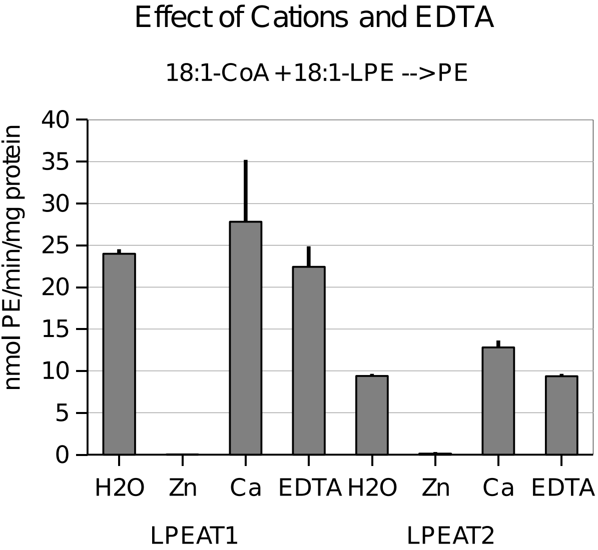

The effect of cations and EDTA on LPEAT1 and LPEAT2 activities. The assays were performed by pre-incubating; 2 mM $ZnCl_2$; 2 mM $CaCl_2$ or 5 mM EDTA with 1.5 µg microsomal protein of yeast (*ale1* strai*n*) from transformants expressing either LPEAT1 or LPEAT2 for 10 minutes at ambient temperature prior to the addition of the enzyme substrates. Error bars indicate standard error of the sample means of triplicate measurements.
